# Watson Abridged: A Synopsis of the Lectures on the Principles and Practice of Physic, Delivered at King’s College, London

**Published:** 1867-04

**Authors:** 


Watson Abridged: A Synopsis of the Lectures on the Principles and Practice of Physic, delivered at King's College, London. By THOMAS WATSON, M.D., etc. (Abridged from the last English Edition,) with a concise but complete Account of the Properties, Uses, Preparations, Doses, etc., of all the Medicines mentioned in these Lectures, and with other valuable additions. By J. J. MEYLOR, A.M., M.D. Philadelphia: Published by the Author. 1867. pp. 277.

This appears to be a faithful abridgement of Dr. Watson's well-known work on the Practice of Physic. It is printed on small but plain type, and supplied with such marginal headings as to facilitate references to particular topics. The author says, in his preface:—"The principal object that induced the making of this abridgement was, to afford young practitioners, who are often at a loss what to do on their first 'sick call,' and other physicians, whom numerous professional duties prevent from consulting more extended works, a convenient and expeditious means of reference in their daily rounds. Another, but no less important object, was to supply medical students, during the lecture seasons, with a ready means of reading over the various subjects treated of in his daily lecture by the Professor on Practice."

